# Language Ability Accounts for Ethnic Difference in Mathematics Achievement

**DOI:** 10.3389/fpsyg.2022.929719

**Published:** 2022-07-22

**Authors:** Jiaxin Cui, Liting Lv, Huibo Du, Zhanling Cui, Xinlin Zhou

**Affiliations:** ^1^College of Education, Hebei Normal University, Shijiazhuang, China; ^2^State Key Laboratory of Cognitive Neuroscience and Learning, Faculty of Psychology, Beijing Normal University, Beijing, China

**Keywords:** ethnic minority, ethnic difference, mathematical achievement, language ability, mathematical cognition

## Abstract

The mathematics achievement of minority students has always been a focal point of educators in China. This study investigated the differences in mathematics achievement between Han and minority pupils to determine if there is any cognitive mechanism that can account for the discrepancy. We recruited 236 Han students and 272 minority students (including Uygur and Kazak) from the same primary schools. They were tested on mathematics achievement, language abilities, and general cognitive abilities. The results showed that Han pupils had better mathematics achievement scores and better Chinese language ability than minority students. After controlling for age, gender, and general cognitive abilities, there were still significant differences in mathematics achievement between Han and minority students. However, these differences disappeared after controlling for language ability. These results suggest that the relatively poor levels of mathematics achievement observed in minority students is related to poor Chinese language skills.

## Introduction

Although numerous cross-cultural studies have been conducted in juveniles, including those on delinquency (e.g., Link, 2008; Kemme, 2010; Schlesinger, 2010; Ungvary et al., 2017; Kobayashi and Farrington, 2019; Yun and Cui, 2019; Mancinelli et al., 2021), cognitive emotions (e.g., Pethtel and Chen, 2010; Alonso-Arbiol et al., 2011; Benítez et al., 2016; Potthoff et al., 2016; Susino and Schubert, 2016; Han et al., 2021; Zhou et al., 2021), traits (e.g., Adams and Hanna, 2012; Feilhauer et al., 2012; Wu and Bodigerel-Koehler, 2013; Bartel-Radic and Giannelloni, 2017; Wilson et al., 2017; Kuśnierz et al., 2020; Srirangarajan et al., 2020), and mental diseases (e.g., Lus and Mukaddes, 2009; Mpango et al., 2017; Wong et al., 2017; Nielsen et al., 2018; Sørlie et al., 2018; Ghafoor et al., 2019), there are relatively few cross-ethnic studies. China is a multi-ethnic country composed of 56 ethnic groups within which about 120 million are ethnic minorities. Among them, 18 ethnic minorities have their own written languages. Therefore, with such a large family of nationalities, cross-ethnic research is very necessary and important in China. Effective cross-ethnic research will help deepen the understanding of the status quo for ethnic differences and allow us to see the challenges that currently exist. These kinds of studies will help narrow the developing gap in education between ethnic minority regions of China and others. Thus, it will help all ethnic groups make common progress (Chen and Xu, 2018, in Chinese version; Hu and Zhou, 2018, in Chinese version).

Previous cross-ethnic studies have focused on social issues, such as social relationships (including neighborhood, e.g., Matlasz et al., 2020), school characteristics (Shin, 2012; Raccanello et al., 2020), classmate relationships (Stark et al., 2015), mental health (Richter et al., 2011; Bhui et al., 2012; Priest et al., 2014), friendship (Munniksma and Juvonen, 2012; Chen and Graham, 2015), medical care and nutrition (including dental care, Vitamins, obesity, and inflammation, Gorelick et al., 2011; Kant and Graubard, 2011; Özel et al., 2020; Scheidell et al., 2020), parental activity (Ali and Frederickson, 2010; Wilson et al., 2011; Cardel et al., 2012), and addiction (including drug, alcohol, and tobacco, e.g., Cummings et al., 2011; Bares and Andrade, 2012; Cook and Caetano, 2014; Greene et al., 2014; Greene and Maggs, 2020). However, past cross-ethnic studies have paid little attention to problems related to academic achievement. In fact, there are often obvious ethnic differences in academic performance. Most previous studies have found that academic performance of ethnic minorities is poorer than that of the majority nationality. This has been shown for African Americans in the United States (Gonzales et al., 2004; Lemke et al., 2004; Perie et al., 2005; Reyna and Brainerd, 2007; Powell et al., 2012; Baxter et al., 2013), and several Indian minorities in India (Jia, 2007, in Chinese version; Yang, 2009, in Chinese version; An and Cheng, 2012, in Chinese version; Wang and Yang, 2019, in Chinese version). As a multi-ethnic country, China also struggles in achieving equal academic performance for its minority populations. Minority students typically lag behind Han Chinese in mathematics achievement during the compulsory education period (Zeng et al., 2015, in Chinese version; He and Sun, 2019, in Chinese version; Liu et al., 2019, in Chinese version).

### Ethnic Differences in Mathematical Achievement

The underachievement of minority students is a worldwide problem. Among them, the poor performance in mathematics has become a prominent problem.

In Europe and America, math scores for minority students differ from those for majority students (Baxter et al., 2013; Sonnenschein and Sun, 2016; Spörlein and Schlueter, 2018; Saw et al., 2018). For example, in a study combining data from 61 countries, the school math test scores of minority students were lower than those of majority students, and minority students tended to score at the lower end of the math spectrum (Spörlein and Schlueter, 2018). Specifically, studies showed that Hispanic children from three urban middle schools scored lower in math than their non-Hispanic peers (Safavian and Conley, 2016), and that ethnic differences begin as early as kindergarten and continue through high school (e.g., Muller et al., 2001; Schneider and Saw, 2016). A study of grades 9–11, for example, also found that Hispanic students scored lower on mathematical assessment tests than White students (about 0.4 standard deviation, Saw et al., 2018). Ethnic differences also exist in some specific mathematical abilities. White children have significantly higher scores in mathematics than Black or Latino children in America, which covers number sense, spatial sense, algebra, and functions (Sonnenschein and Sun, 2016). Similarly, the Program for International Student Assessment found that in many Organization for Economic Co-operation and Development (OECD) countries (e.g., France, Switzerland, and the United Kingdom), immigrant student performance was not as good as that of native students in both reading and mathematics (OECD, 2010). Another study compared the math achievement gap between 4th grade mainstream students and immigrant students in TIMSS 2015, and showed that for all countries, 4th grade immigrant students performed significantly lower than mainstreamers (*p* < 0.001), except in England and Ireland (Arikan et al., 2020).

As a developing country with a large population, India’s minority education issues are very prominent (Karuna, 1993; Arouri et al., 2019). The Scheduled Castes and Tribes account for about 15% of the Indian population (Das, 1982; Gallanter, 1984), and are usually regarded as minorities in the sociological literature (Vander Zanden, 1983). In India, the low social status and remote living areas of the Scheduled Castes and Tribes have increased the difficulty of providing adequate literacy education, resulting in literacy rates that are far lower than the national average (Huang, 1998, in Chinese version). The gap in absolute illiteracy between Scheduled Tribes and non-Scheduled Tribes rose from 22% in 1971 to 33% in 1991, before falling to 18% in 2001 (Ministry of Tribal Affairs, 2008). In 1999, the Indian Scheduled Tribes and non-Scheduled Tribes had a secondary educational completion rate of 4.35 and 21.99%, respectively, and by 2009, Scheduled Tribe’s data had risen to 7.98%, But far less than the 32.8% of Non-Scheduled Tribe. After more than 10 years of development, the difference between Scheduled Tribes and non-scheduled Tribes is not narrowing, but widening (Basant and Sen, 2014). Arouri et al. (2019) studied the disparities in ethnic and racial education in India and found that academic performance of ethnic minorities is lower than that of the main ethnic groups in all respects, including percentage of being enrolled in school, grade of education completed, and math test scores, to name a few.

Ethnic differences in mathematical achievement are also prominent in China (Zeng et al., 2015, in Chinese version; Liu et al., 2019, in Chinese version). Studies have found that the standard math scores of minority students are significantly lower than those of Han students (e.g., Zeng et al., 2015, in Chinese version). There is also a significant difference in passing rate for mathematics between minority students and Han students; the passing rate on the final mathematics examination has been lower for ethnic minority students (79.5%) than for Han students (87.5%) in 2019 (Liu et al., 2019, in Chinese version). In order to alleviate this problem, the Chinese government has carried out a series ethnic education initiatives in terms of legislation, policy formulation, and training plans (Chen and Zhong, 2018, in Chinese version; Ren and Wei, 2018, in Chinese version). The Fourteenth Five-Year Plan for the National Economic and Social Development and the Outline of the Vision to 2035 adopted at the Fourth Session of the Thirteenth National People’s Congress of China both put forward the requirements of “improving the quality and level of education in ethnic minority areas and strengthening the promotion of the national common language.”

### Reasons for Ethnic Differences in Math Achievement

Previous studies have primarily investigated ethnic differences in mathematical ability from the perspective of social factors, such as socioeconomic status, educational resources, parental involvement, and emotional factors (Azmitia et al., 2008; Halpern-Manners et al., 2009; Ceballo et al., 2014; Sonnenschein and Sun, 2016; Assari et al., 2020; Brandmiller et al., 2020).

Ethnic differences in academic achievement often result from differences in socioeconomic status (Sonnenschein and Sun, 2016; Assari et al., 2020). Family poverty is one of the strongest barriers to educational success for an ethnic minority student (Dahl and Lochner, 2012; Trieu and Jayakody, 2018; Dolean and Cãlugãr, 2020). For example, ethnic minority groups perform worse than White Americans on math scores in American (Potter and Morris, 2016) and tend to be poorer than White Americans in terms of economic status (Dahl and Lochner, 2012; Trieu and Jayakody, 2018). A study of Romania children found that living conditions could partially mediate the relationship between ethnicity and IQ (Dolean and Cãlugãr, 2020). The educational level of parents is another effective indicator of socio-economic status. Lower academic achievement levels of minority students are usually closely related to the lower educational level of their parents (Dolean and Cãlugãr, 2020). Similarly, a study of African Americans and White Americans in the United States has reported that higher parental education is associated with higher math and reading scores (Assari et al., 2020).

Inequality between minorities and the majority in educational resources, such as school quality and learning environment also contributes to ethnic differences in mathematics (e.g., Halpern-Manners et al., 2009). Differences in the quality of schools attended lead to differences in academic achievement between White Americans and African Americans or Hispanic Americans (Halpern-Manners et al., 2009). In Vietnam, being closer to school as well as effective support from parents, relatives, schools, and school peers was reported to effectively prevent minority students from dropping out of school and to promote academic success (Trieu and Jayakody, 2018).

The lack of parental involvement in education is also a contributing factor to poor academic ability in ethnic minority students. Some longitudinal studies have found that Asian and White children perform better than Black and Hispanic children in math and reading in United States, which was mediated by parental awareness and involvement in the their children’s educational development (Huang et al., 2005; Azmitia et al., 2008; Ceballo et al., 2014; Sonnenschein and Sun, 2016). Another study in the southwestern United States showed that sensitive support from African American and Hispanic American fathers was associated with their children’s mathematics achievement, even after controlling for sensitive support from the mothers (Caughy et al., 2020).

Social emotional factors, such as motivation and attitudes, also contribute to ethnic differences in math achievement. The motivation to learn mathematics for minority students is not clear. Brandmiller et al. (2020) showed that learning motivation was significantly correlated with immigrant backgrounds (*r* = 0.09, *p* < 0.001) and that learning motivation was less in students with immigrant backgrounds than in students without immigrant backgrounds. There were also significant differences between Han and Tibetan students in math knowledge memory, math logical thinking, the will to learn math, and math learning motivation (*p* < 0.005), among which, the factor with greatest difference was the motivation to study math (Xi and Zhang, 2008, in Chinese version). Learning attitudes for minorities are relatively weak. Trieu and Jayakody (2018) suggested that rejection of the official language by minority Vietnamese students is a major obstacle to their learning, and the social distance between majority teachers and minority students may weaken their bond (Giacchino-Baker, 2007; Baulch et al., 2010). Many researchers have found that minority students in Vietnam rarely interact with their teachers, they usually sit quietly in class and do not participate in class discussions (Tran, 2013), which are all manifestations of the negative learning attitude of minority students.

To sum up, minority students in many countries generally face the problem of poor performance in mathematics, and most existing studies have tried to explain such ethnic differences by social factors, such as SES, education resources, parental involvement in education, or social emotion (Klebanov et al., 1998; Dahl and Lochner, 2012; Sonnenschein and Sun, 2016; Assari et al., 2020).

We think there is another possible explanation for the difference in mathematical ability between the majority and ethnic minorities. Ethnic minorities often use their own national language as their mother tongue, which might prevent adequate fluency in a country’s official language. However, there no study has examined whether differences in language processing ability alone can account for ethnic differences in mathematical achievement.

### Current Investigation

Different from previous studies that focused on socioeconomic status, educational resources, parental involvement, or social emotions, the current study explored the cognitive mechanisms for the ethnic differences in mathematical ability. As far as we know, this is the first study to investigate this question. We hypothesized that processing abilities for an official language (in this case Chinese) could fully account for the ethnic differences in mathematics achievements. Language competence in this study refers to the ability to use the official written language of one’s own country.

The reason for this hypothesis is that language is an important resource in mathematics education (e.g., Barwell, 2018; Planas, 2018), and mathematics knowledge is transmitted to students through language expression. Therefore, teaching in different languages may bring different effects, and different language environments may bring students different priori knowledge and cognitive reserves. Two aspects of empirical evidences can indirectly support this view.

First, crucial language elements including phonetics and semantics are involved in math processing. For example, representation of mathematical terms and operational symbols requires coding of phonetic information (Hecht et al., 2001; Simmons and Singleton, 2008). Reading comprehension and mathematics is highly correlated in students [*r* = 0.55, see a review by Singer and Strasser (2017)]. Evidence from brain lesion studies indicate that serious semantic deficits due to atrophy in the left temporal cortex are accompanied by deficits in arithmetical computation and principle understanding (Julien et al., 2008; Butterworth et al., 2011).

Second, studies have found that students whose mother tongue is not the national language of a country, or the language used in broader educational communication, usually exhibit poorer academic performance. For example, language barriers were among the major factors for the relative low math performance of non-English-speaking minority groups in the United States (Serratrice et al., 2006; August et al., 2009). The differences in math achievement between monolingual German students and German students who spoke a linguistic minority language were related to differences in their language levels (Haag et al., 2014). In Vietnam schools, linguistic barriers can also decrease educational achievement and attainment for ethnic minorities (Taylor, 2007). Similar phenomena also appear in China. Ethnic minority students in western China, especially those whose mother tongue is not Mandarin, scored significantly lower on standardized math tests than did Han students (0.62 standard deviations lower, see Yang et al., 2015). In math class, students taught in Chinese language performed better than those taught in Mongolian (*p* < 0.01, see Zhong, 2013, in Chinese Version). Moreover, when both had Chinese as their classroom language, students whose at-home language was Chinese performed better than those whose at-home language was Mongolian (*p* = 0.026; Zhong, 2013, in Chinese Version).

In order to study the influence of language ability on ethnic differences in mathematics, we controlled for the influence of socioeconomic status factors and educational factors on mathematical achievement. We selected Han and ethnic minority students as participants from several schools, with the same number of Han and ethnic minority students being selected from each school. Because of the proximity policy, these students live in the same or adjacent blocks and thus have similar socioeconomic status. At the same time, they study in the same schools and receive the same education, which controls for the influence of educational factors. In addition, we controlled for some general cognitive variables related to math, such as reaction time, general intelligence, and spatial ability.

## Materials and Methods

### Participants

Participants were 508 Han and minority Chinese students in elementary schools (222 fourth graders and 286 fifth graders). The fourth graders (106 male and 116 female; 125.64 ± 9.5 months) comprised 118 Han students (64 male and 54 female) and 104 minority students (42 male and 62 female) and the fifth graders (152 male and 134 female; 138.23 ± 8.6 months) comprised 118 Han students (72 male and 46 female) and 168 minority students (80 male and 88 female).

All children were recruited voluntarily from public inner-city primary school in Xinjiang province. Each elementary school has roughly the same number of Han students and minority students, and each class of these schools has both minority and majority students. Therefore, all children should have equal access to the same education. The minority children (including Uygur and Kazak) are bilingual (with Uyghur or Kazakh as their first language, respectively), and have been exposed to bilingual environment since birth (Tsung and Cruickshank, 2009). They began their formal learning of Chinese since the preschool education stage, and their compulsory education stage is completely in the educational environment with the National common language as the communication medium (Jia, 2020, in Chinese version). All children had normal or corrected-to-normal vision, and no children were diagnosed with intellectual, behavioral or sensory impairments. Obtain parental consent before the classroom testing.

### Tests

**FIGURE 1:**
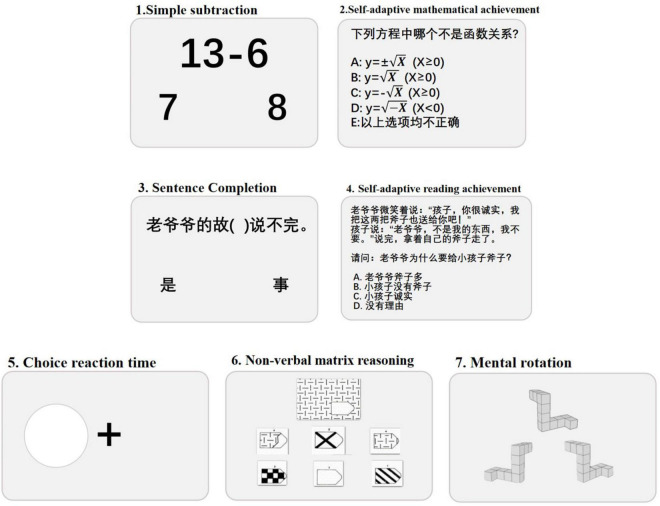
Schematic representation of tests used in the current study.

A total of seven tests were used. All tests were implemented through web-based applications in the Online Psychological Experiment System (OPES)^1^. Figure 1 shows the schematic of these seven tests. In addition to the self-adaptive mathematical achievement and self-adaptive reading achievement tests, each test had a training session followed by an official testing session. Six of these tests have been reported formerly (Zhou et al., 2015; Wang et al., 2016; Cui et al., 2019) and they displayed acceptable half-split reliabilities, ranging from 0.80 to 0.96. The self-adaptive reading achievement test is the only test we used that has not been previously reported. However, its design is essentially identical to that of the self-adaptive mathematical achievement test. Therefore, we speculate that it would also have an acceptable half-split reliability.

#### Simple Subtraction

This test is used to measure arithmetic fluency. It included 92 single-digit subtraction problems. A subtraction problem (e.g., 13–6) was appeared on the screen, with two candidate response displayed underneath. Every minuend ranged from 2 to 18, and answers ranged from 2 to 9. The incorrect choice deviated from true answer by ±3. The formal testing was restricted to 2 min.

The adjusted number of correct trials was utilized to control for the impact of speculating in multiple choice tests. The test grade was determined by deducting the quantity of erroneous reactions from the quantity of right reactions following the Guilford correction formula: *S* = *R* – *W*/(*n* – 1), where S is the adjusted quantity of correct items without the help of possibility, *R* is the quantity of correct reactions, *W* is the number of mistakes, and *n* is the quantity of alternative responses for every item (Guilford, 1936). This correction methodology has been used as of late in researches of mathematical cognition (Cirino, 2011; Zhou et al., 2015; Cui et al., 2019) and cognition in general (Salthouse, 1994; Putz et al., 2004; Hedden and Yoon, 2006).

#### Self-Adaptive Mathematical Achievement

This test was used to measure overall mathematical achievement. It was self-adapting and had a time limit of 18 min. The questions were chosen from typical exercise books and test papers used by Chinese students, which were related to the school textbooks that were used by students throughout their education (12 grades: 6 in primary school, 3 in junior high school, and 3 in senior high school). All questions were mathematical problems of graded difficulty levels. For the primary school problems, the questions were relatively easy to solve [e.g., () + 7.4 = 16; (a) 4, (b) 5, (c) 6, (d) 7. Junior high school level questions were harder to solve [e.g., Which of the following is *NOT* a functional relationship? (a) $y\pm \sqrt{X}\left(x\geq 0\right)$, (b) $y=\sqrt{X}\left(x\geq 0\right)$, (c) $y=-\sqrt{X}\left(x\geq 0\right)$, (d) $y=\sqrt{-X}\left(x<0\right)$, (e) none of the above]. Additional examples can be seen in Figure 1. Problems for senior high school students were the hardest to solve (e.g., If the set A = 1, 2, 3, 4, B = x| x = n^2^, n∈A, then A∩B = (); (a) {1,4}, (b) {2,3}, (c) {9,6}, (d) {1,2}). The questions for each grade were randomly chosen and grouped into five sets. Each set included three questions that came from different chapters in the textbooks. The experimental procedure was as follows. Participants were first given a set of questions from the first grade. For each set, if they correctly solved at least two of the three problems, the difficulty level advanced to the next grade, if they answered two of the three problems incorrectly, the difficulty level remained at the same grade, and if they answered all three questions incorrectly, the difficulty level dropped a grade (unless it was already at the first grade). In addition, if participants failed to solve all five sets of questions in a grade, the difficulty level dropped a grade. The test stopped at the end of the allocated time or if all five sets of questions in the current grade were completed. The final score was calculated as the average value of the sum of weighted scores in each grade, which was the number of correctly answered questions multiplied by the grade level (1–12). A total of 1,701 problems were included in our test database.

#### Sentence Completion

The task resembled which utilized by previous research (So and Siegel, 1997) and was utilized to estimate reading comprehension fluency (e.g., Elbeheri et al., 2011; Träff et al., 2018). Materials of the task were based on the test materials utilized in Chinese schools. The vocabulary included in this test and the next one is all chosen according to the national curriculum standards of China to ensure that minority students will not encounter strange scenes and words. Moreover, the language materials are related to the content of language learning, but not to the content of national culture. For each experiment, a sentence with one word missing was appeared in in the middle of the computer screen. Participants required to choose one of the two candidate words at the bottom of the screen to fill the sentence. There were 120 questions, set from simple to hard. Participants need to choose answers fast and precisely, and to finish the trials as many as possible. The task’s formal test was restricted to 5 min.

#### Self-Adaptive Reading Achievement

Self-adaptive reading achievement was used to assess Chinese language comprehension ability. The test was self-adapted and had a time limit of 20 min. Task rules and procedures were the same as the self-adaptive math achievement test. The questions in the test mainly come from the school’s final exams of each semester, testing the students’ language ability, including language knowledge, understanding, discrimination and so on (e.g., The old grandpa smiled and said “Kid, you are honest. I will give you these two axes as well”). The child said: “old grandpa, they are not mine, I don’t want these,” then the child went away with his axe. The question: why does the old grandpa want to give the child axes? (a) The old grandpa has many axes, (b) The child doesn’t have axes, (c) The child is honest, (d) It has no cause.

#### Choice Reaction Time

The basic reaction time task was used to control for the influence of manual reaction and psychological processing speed. For each trial, a fixed cross is placed in the center of the screen. After fixating the cross for a period of time, a white dot, appeared on the left or right of the fixed cross. Participants were asked to press a key on the keyboard as soon as the white dot appeared. When the dot appeared on the left, they pressed “Q,” and when the dot appeared on the right, they pressed “P.” There were 30 trials. The inter-trial interval was chosen between 1,500 and 3,000 ms randomly.

The median reaction time and error rate were recorded, but the overall average error rate of the selected response time task was very low (4.72%), so we didn’t further analyzed.

#### Non-verbal Matrix Reasoning

Nonverbal matrix reasoning was utilized to assess general IQ. The test was adapted from Raven’s Progressive Matrices (Raven, 2000). A picture with a missing section was presented to the participants. The task was to choose the missing portion of the image from six candidate images. The test had 36 trials and stopped when five total trials were incorrect. The number of correct trials was used as the test score.

#### Mental Rotation

The mental rotation test was based from Shepard and Metzler (1971). The modified version had only two choices, and the time limit is 3 min. Each trial had three 3-D images: one of the images was at the top of the screen and the remaining two were at the bottom of the screen. Participants were asked to do a mental spin and decide which of the following two choices was the same as the one above. Rotated the image from the original image with a rotation angle ranging from 15° to 345° (at intervals of 15°). The other image was a mirror image of the target. If the selection was on the left, participants press the “Q” key and if the selection was on the right, participants press the “P” key. The adjusted number of correct trials was utilized as the test score (see the simple subtraction test).

### Procedure

In the computer classroom, students (one class at a time) perform computerized tasks. Each class was supervised by two or three experimenters and the teacher of the class. The experimenter used slides to explain the description of each task. The teacher was present only for the purpose of discipline (e.g., remaining silence during the formal testing). After all students completed a test, the experimenter began to manage the following test. For each test, students were first instructed to complete an training session, and then continued the formal test. The training test contained 4–6 trials that were similar to those used in the formal test. In the training stage, for correct answers the feedback of all tasks was “Right! Can you do faster?”, and for incorrect answers was “Incorrect, another attempt, and you’ll succeed.” The formal test began after all the students completed the training stage. When all students understood the procedure during training stage, they could start the formal test. When the experimenter said “Go ahead!”, all students press a key to start the formal test. They all carry out their tasks in the same order. Responses and reaction times were automatically recorded.

### Data Analysis

Descriptive statistics and pairwise correlations (with Bonferroni correction) were obtained for all tasks. Then, a series of multivariate analyses were carried out to test the difference in mathematics ability between Han majority and ethnic minority Chinese elementary school students. A stratified regression analysis was then performed, controlling for age (the variation in months within an age group), gender, three kinds of general cognitive processes (choice reaction time, non-verbal matrix reasoning, mental rotation), sentence completion, and self-adaptive reading achievement.

## Results

Table 1 shows the means and standard deviations of scores and the ethnic differences for all seven tasks in the research.

**TABLE 1:** Descriptive statistics for all the measures used in the study.

Grade	Tests	Indices	Han mean (SD)	Minority mean (SD)	*F-*value	*P-*value	η^2^
4	Simple subtraction	Adjusted number of correct trials	34.9 (9.4)	28.2 (11.6)	21.93	<0.001	0.09
	Self-adaptive mathematical achievement	Score	11.2 (5.0)	8.0 (4.4)	25.14	<0.001	0.11
	Sentence completion	Adjusted number of correct trials	23.5 (8.0)	9.7 (10.9)	114.33	<0.001	0.35
	Self-adaptive reading achievement	Score	10.2 (8.7)	4.9 (5.6)	27.80	<0.001	0.12
	Choice reaction time	Reaction time (seconds)	449 (104)	549 (164)	29.89	<0.001	0.12
	Non-verbal matrix reasoning	Number of correct trials	4.6 (2.7)	4.4 (2.5)	0.33	0.57	0.00
	Mental rotation	Adjusted number of correct trials	10.5 (10.7)	8.3 (11.1)	2.31	0.13	0.01
5	Simple subtraction	Adjusted number of correct trials	35.0 (12.3)	29.2 (11.8)	16.40	<0.001	0.06
	Self-adaptive mathematical achievement	Score	13.3 (5.7)	8.8 (4.4)	57.89	<0.001	0.17
	Sentence completion	Adjusted number of correct trials	24.1 (9.6)	11.9 (11.9)	85.04	<0.001	0.23
	Self-adaptive reading achievement	Score	11.5 (9.1)	4.5 (5.0)	69.12	<0.001	0.20
	Choice reaction time	Reaction time (seconds)	429 (109)	506 (149)	22.81	<0.001	0.08
	Non-verbal matrix reasoning	Number of correct trials	5.2 (2.9)	4.0 (2.6)	17.35	<0.001	0.06
	Mental rotation	Adjusted number of correct trials	12.1 (12.1)	11.3 (10.9)	0.39	0.534	0.00

*These are the values before conducting any transformations. Adjusted number of correct trials = Total correct trials minus total incorrect trials. This adjustment was made to control for the effect of guessing on multiple choice tests.*

Table 2 reveals the correlations between all measures used in the research, with minority Chinese above the diagonal and Han Chinese below the diagonal. Bonferroni correction (*p*-value × 21) was used adjust for the 21 comparisons. A corrected *p*-value < 0.05 was considered statistically significant.

**TABLE 2:** Correlations between the measures used in the study for Minority (above the diagonal) and Han (below the diagonal).

Grade		1	2	3	4	5	6	7
4	1. Simple subtraction	−	0.42*	0.44*	0.10	−0.30*	0.29	0.28*
	2. Self-adaptive mathematical achievement	0.34*	−	0.29	0.29	−0.05	0.35*	0.22*
	3. Sentence completion	0.44*	0.27	−	0.47*	−0.27*	0.29	0.13
	4. Self-adaptive reading achievement	0.43*	0.57*	0.45*	−	−0.15	0.10	−0.09
	5. Choice reaction time	−0.12	−0.18	−0.13	−0.21	−	−0.22	−0.17
	6. Non-verbal matrix reasoning	0.20	0.30*	0.19	0.35*	−0.20	−	0.28
	7. Mental rotation	0.15	0.08	0.12	0.10	−0.01	0.05	−
5	1. Simple subtraction	−	0.35*	0.19	0.13	−0.29*	0.34*	0.22
	2. Self-adaptive mathematical achievement	0.40*	−	0.41*	0.49*	−0.14	0.35*	0.19
	3. Sentence completion	0.43*	0.40*	−	0.37*	−0.26*	0.21	0.17
	4. Self-adaptive reading achievement	0.30*	0.40*	0.34*	−	−0.13	0.23*	0.15
	5. Choice reaction time	−0.06	0.13	−0.03*	−0.03	−	−0.23	−0.17
	6. Non-verbal matrix reasoning	0.26	0.30*	0.29*	0.23	−0.12	−	0.21
	7. Mental rotation	0.40*	0.36*	0.19	0.07	0.10	0.25	−

*p < 0.05, Bonferroni-corrected.

Table 3 reveals the partial correlations between all measures used in the research, with minority Chinese above the diagonal and Han Chinese below the diagonal (controlling for age and gender). Bonferroni correction (*p*-value × 21) was used adjust for the 21 comparisons. A corrected *p*-value < 0.05 was considered statistically significant. The relationships between measures for mathematics achievement and language ability were consistent across the two grades (4th and 5th), indicating stability over time.

**TABLE 3:** Partial correlations between the measures used in the study for Minority (above the diagonal) and Han (below the diagonal) (controlling for age and gender).

Grade		1	2	3	4	5	6	7
4	1. Simple subtraction	−	0.48*	0.42*	0.12	−0.24	0.28	0.18
	2. Self-adaptive mathematical achievement	0.33*	−	0.29	0.25	−0.13	0.33*	0.18
	3. Sentence completion	0.43*	0.24	−	0.31*	−0.41*	0.29	0.12
	4. Self-adaptive reading achievement	0.46*	0.56*	0.45*	−	−0.18	0.12	−0.04
	5. Choice reaction time	−0.15	−0.20	−0.15	−0.17	−	−0.25	−0.14
	6. Non-verbal matrix reasoning	0.21	0.32*	0.20	0.36*	−0.20	−	0.25
	7. Mental rotation	0.13	0.09	0.12	0.15	−0.01	0.06	−
5	1. Simple subtraction	−	0.35*	0.19	0.15	−0.24*	0.35*	0.24*
	2. Self-adaptive mathematical achievement	0.37*	−	0.41*	0.49*	−0.16	0.35*	0.20
	3. Sentence completion	0.38*	0.36*	−	0.37*	−0.26*	0.21	0.18
	4. Self-adaptive reading achievement	0.22	0.37*	0.26	−	−0.16	0.24*	0.15
	5. Choice reaction time	−0.14	0.01	−0.05	−0.09	−	−0.27*	−0.22
	6. Non-verbal matrix reasoning	0.29*	0.33*	0.32*	0.27	−0.09	−	0.22
	7. Mental rotation	0.45*	0.39*	0.24	0.14	0.09	0.24	−

*p < 0.05, Bonferroni-corrected.

Table 4 shows the contribution of ethnicity to ethnic differences after controlling for age, gender, and the three general cognitive factors. The two sets of data display consistent results for children in fourth and fifth grade, again indicating that ethnic differences are stable over time.

**TABLE 4:** Hierarchical regression models predicting simple subtraction and self-adaptive mathematical achievement from age, gender, general cognitive processing, and ethnicity.

Grade	Predictors	Simple subtraction			Self-adaptive mathematical achievement		
		Step 1 Beta	Step 2 Beta	Step 3 Beta	Step 1 Beta	Step 2 Beta	Step 3 Beta
4	Age (months)	−0.10	−0.04	−0.02	−0.05	0.01	0.03
	Gender	−0.10	−0.00	0.00	0.10	0.20*	0.23*
	Choice reaction time	−	−0.24*	−0.16	−	−0.19*	−0.08
	Mental rotation	−	0.15	0.14	−	0.10	0.09
	Non-verbal matrix reasoning	−	0.17	0.18*	−	0.26*	0.27*
	Ethnicity	−	−	−0.23*	−	−	−0.31*
		*R*^2^=0.004	*ΔR^2^*=0.133*	*ΔR^2^*=0.044*	*R*^2^=0.015	*ΔR^2^*=0.140*	*ΔR^2^*=0.082*
5	Age (months)	0.02	0.03	0.03	−0.13	−0.11	−0.10
	Gender	0.10	0.10	0.20	0.00	0.10	0.10
	Choice reaction time	−	−0.19*	−0.16*	−	−0.10	−0.03
	Mental rotation	−	0.25*	0.26*	−	0.18*	0.20*
	Non-verbal matrix reasoning	−	0.25*	0.22*	−	0.33*	0.26*
	Ethnicity	−	−	−0.15*	−	−	−0.35*
		*R*^2^ = −0.004	*ΔR^2^* = 0.213*	*ΔR^2^* = 0.020*	*R*^2^ = 0.012	*ΔR^2^* = 0.195*	*ΔR^2^* = 0.108*

*p < 0.05, Bonferroni-corrected.

Table 5 shows the unique contribution of language ability to ethnic differences after controlling for demographic variables (age and gender), the three general cognitive factors, the two types of language ability (self-adaptive reading achievement and sentence completion), and ethnicity. The two panels show consistent results for Han children and minority children in fourth and fifth grade; after controlling for language, ethnic differences in math scores cease to exist.

**TABLE 5:** Hierarchical regression models predicting simple subtraction and self-adaptive mathematical achievement from age, gender, general cognitive processing, sentence completion, self-adaptive reading achievement, and ethnicity.

Grade	Predictors	Simple subtraction				Self-adaptive mathematical achievement			
		Step 1 Beta	Step 2 Beta	Step 3 Beta	Step 4 Beta	Step 1 Beta	Step 2 Beta	Step 3 Beta	Step 4 Beta
4	Age (months)	−0.10	−0.04	−0.02	−0.02	−0.05	0.01	0.03	0.04
	Gender	−0.10	−0.00	−0.10	−0.10	0.10	0.20*	0.10	0.10
	Choice reaction time	−	−0.24*	−0.05	−0.06	−	−0.19*	−0.04	−0.03
	Mental rotation	−	0.15	0.10	0.10	−	0.10	0.07	0.07
	Non-verbal matrix reasoning	−	0.17	0.10	0.09	−	0.26*	0.16	0.18*
	Sentence completion	−	−	0.39*	0.40*	−	−	0.16	0.09
	Self-adaptive reading achievement	−	−	0.13	0.13	−	−	0.39*	0.37*
	Ethnicity	−	−	−	0.02	−	−	−	−0.15
		*R*^2^=0.013	*ΔR^2^*=0.133*	*ΔR^2^*=0.165*	*ΔR^2^*=0.000	*R*^2^=0.024	*ΔR^2^*=0.140*	*ΔR^2^*=0.185*	*ΔR^2^*=0.013
5	Age (months)	0.02	0.03	0.04	0.04	−0.13	−0.11	−0.10	−0.10
	Gender	0.10	0.10	0.10	0.10	0.00	0.10	0.00	0.00
	Choice reaction time	−	−0.19*	−0.13	−0.13	−	−0.10	0.03	0.03
	Mental rotation	−	0.25*	0.23*	0.23*	−	0.18*	0.14*	0.15*
	Non-verbal matrix reasoning	−	0.25*	0.18*	0.18*	−	0.33*	0.17*	0.16*
	Sentence completion	−	−	0.17	0.15	−	−	0.28*	0.24*
	Self-adaptive reading achievement	−	−	0.11	0.09	−	−	0.34*	0.30*
	Ethnicity				−0.05				−0.13
		*R*^2^= 0.003	*ΔR^2^* = 0.213*	*ΔR^2^* = 0.044*	*ΔR^2^* = 0.002	*R*^2^ = 0.019	*ΔR^2^* = 0.195*	*ΔR^2^* = 0.226*	*ΔR^2^* = 0.012

*p < 0.05, Bonferroni-corrected.

**FIGURE 2:**
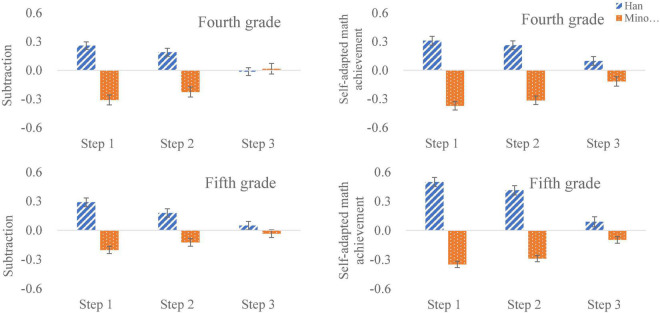
Differences in the mean of the standardized residuals for subtraction and self-adapted math achievement between Han and minority elementary school students for fourth grade (top) and fifth grade (bottom) after controlling for age, gender (Step 1), choice reaction time, non-verbal matrix reasoning, and mental rotation (Step 2), sentence completion, and self-adapted reading comprehension (Step 3).

The regression results in Tables 4, 5 displayed that the ethnic differences in math scores can be explained by language ability (measured by sentence completion and self-adaptive reading achievement); when ethnicity was added to the regression (in the fourth step), it did not significantly contribute to simple subtraction or self-adaptive mathematical achievement for children of any age group. It can also be seen from Figure 2 that the ethnic differences in simple subtraction and self-adaptive mathematical achievement were significantly reduced after controlling for language factors.

## Discussion

The current investigation examined whether skill at the Chinese language, as an official language, could explain mathematical achievement difference among ethnic groups in China. After controlling for age, gender, and general cognitive factors, we found that a significant difference in mathematical processing remained between minority pupils and Han pupils. Further controlling for language processing (measured by sentence completion and self-adaptive reading achievement), these ethnic differences in mathematical achievement disappeared. This means that language ability could account for the ethnic differences in mathematical achievement.

The current study repeatedly verified the findings of most previous studies; academic achievement in ethnic minorities is significantly lower than that of the ethnic majority, and both Chinese and international studies have found similar phenomenon (Potter and Morris, 2016; Sonnenschein and Sun, 2016; Kuhfeld et al., 2018). First, mathematical performance in ethnic minorities is lower, in both overall mathematical achievement and in special types of mathematical ability, such as arithmetic computation and problem solving (Kong and Orosco, 2015; Lonigan et al., 2015; Spörlein and Schlueter, 2018; Saw et al., 2018; Assari et al., 2020). Second, language skills in the county’s official language is lower in ethnic minorities than in the ethnic majority (Potter and Morris, 2016; Sonnenschein and Sun, 2016; Kuhfeld et al., 2018). This is reflected in the poorer ability of ethnic minorities to use the official language to understand mathematical terms and to express mathematical knowledge (Cummins, 1979, 2000; Guan, 2020, in Chinese version).

The current study further found that the degree of mathematical disadvantage observed in ethnic minorities can differ depending on the type of math. In mathematics, the ethnic minority performance was better on simple problems than it was on complex problems. Thus, their disadvantage for calculation ability was less than that for problem-solving. This may be because calculation is relatively simple in that it can be completed through memory retrieval and improved through frequent practice (Calderón, 2016). In contrast, problem solving is more complex and difficult, requires a wide range of knowledge, and a deeper conceptual understanding (Passolunghi et al., 2018; Villeneuve et al., 2019). Supporting this idea, one study found that ethnic minority students at risk for math difficulties struggle with word problems for various reasons beyond procedural or calculation challenges (Kong and Orosco, 2015).

We also found that ethnic differences in language depending on the type of language skill being tested. Unlike the situation with math, we found that the disadvantage for reading comprehension of relatively simple sentences was far greater than that for more complex text. This may be because the sentence itself contains fewer words, so it provides less information. If a student has difficulty in the orthography or semantics of just one or two key words, it will affect understanding of the whole sentence (Scott, 2009; Poulsen and Gravgaard, 2016). When texts are longer and more information is provided, if individual words cannot be understood clearly, the overall meaning might still be understood based on the remaining text. Additionally, students can also use reasoning and other strategies to solve problems on reading comprehension tests (Segers and Verhoeven, 2016).

We confirmed our hypothesis that the low mathematical ability observed in ethnic minorities is caused by their low language ability. This is consistent with the circumstantial evidence found in previous studies (Vukovic and Lesaux, 2013; Wilkinson, 2018). For example, among Latinos, Limited English Proficiency (LEP) has generally been linked to lower achievement outcomes in mathematics (Padilla and Gonzalez, 2001; Eamon, 2005). North American immigrants also performed at lower math levels than non-immigrants, and it is speculated that this may be because immigrants are less proficient in English (Barrett et al., 2012).

The low mathematical ability observed in ethnic minorities might also come from the influence of their mother tongue thinking. Though the word formation of numerals is similar among Chinese, Uyghur and Kazakh languages (Pan, 1982, in Chinese version; Liu et al., 2012, in Chinese Version), there is linguistic difference in word order between Uyghur and Chinese, that is, Uyghur has “subject + object + verb” structure, while has “subject + verb + object” structure. The psychological stereotype of word order of mother tongue would also influence mathematics processing, especially arithmetic. Students sometimes can’t figure out the operation sequence when doing the four operations. For example, when calculating “144–960 ÷ 48”, influenced by their mother tongue, they might first calculate “960–144”, and then “÷ 48”; when fourth-graders reading decimals, they are distracted by this grammatical discrepancy and often pronounce “11.01” as “one zero point one one”, and “0.32” will be read as “twenty-three point zero” (Zhang and Gao, 2016, in Chinese version). The main reason for this is that minority students’ Chinese ability is not proficient enough, which makes them subconsciously choose their own language to deal with mathematical problems. However, mutual interference between the two languages that minority bilinguals master (e.g., Kroll et al., 2014) would cause a slower speed than Han students to solve mathematical problems. This has also been confirmed in previous studies that bilinguals performed lower than first language learners in early calculation (Bonifacci et al., 2016).

The results of this study further support the theory that mathematics and language are highly correlated (Vukovic and Lesaux, 2013; Foster et al., 2018; Schmitt et al., 2019). Previous studies have also speculated that disadvantages in mathematics seen in minority groups are related to their poorer ability to use the official language in the classroom. It is difficult for both teachers and students of minority populations to express mathematical knowledge in the official language. For example, German minority student math scores are usually lower than those of native students. Moreover, math problems with high linguistic requirements are more difficult for students who use native ethnic minority languages, especially when they include special academic vocabulary (such as “denominator”) (Haag et al., 2014). Our research shows that the poor official language ability of ethnic minorities is not only reflected in their mathematical knowledge or the scientific language used in textbooks, but also in the general language ability used in daily life. Therefore, general language ability itself can explain the relatively poorer mathematical ability seen in ethnic minorities.

Though minority students usually has lower scores in language tests, their listening and speaking ability may be far better than their reading and writing ability. Indeed, on the ethnic minority preparatory Chinese level test, their listening comprehension ability is better than their reading comprehension and writing abilities (Guan, 2020, in Chinese version). Previous studies have also reported that ethnic minorities can communicate with others skillfully in official languages and communicate without obstacles in daily life (Yu, 2012, in Chinese version; Shi, 2018, in Chinese version). In China, most ethnic minority students have no problems in daily communication, but their scores on Chinese exams are poor (Yang, 2015, in Chinese version), which indicates that daily Chinese is not exactly the same as the Chinese used on exams or in mathematics. Mathematics usually uses academic language (Haag et al., 2014), which is frequently assumed to be especially challenging for minority students (Heppt et al., 2014). For second-language learners, mastering academic language is also expected to be more challenging than mastering daily language (Cummins, 1979, 2000). Their lack of communication in the classroom could be due to the lack of scientific and mathematical vocabulary in their native minority languages, whereas the vocabulary of everyday life is generally translatable between all languages. This shows that the development of the two functions of language as a tool of social communication and as a vehicle of imparting knowledge may be asynchronous.

Although relatively poor official language ability of ethnic minority students may affect their math performances, this does not mean that they should abandon their national language. Minority students are generally able to master multiple languages, and making good use of their multilingual advantages in mathematics education may be a more economical and efficient method (e.g., Erath et al., 2021). Prediger et al. (2019) provided an example of multilingual resources to help seventh graders deepen their concept understanding of fractions. In short, language should be the perfect carrier for establishing fair and high-quality mathematics education and teaching.

This study also examined the general cognitive level of ethnic minorities and as expected, found it does not significantly differ from that of ethnic majority groups. This could be because education has a limited impact on general cognitive ability, or it could be that the education minorities receive is sufficient to support normal cognitive development. This study cannot verify which hypothesis is correct, but nevertheless, we can say that intelligence levels between ethnic minorities and the majority are comparable and cannot explain differences in mathematical achievement.

The current study also found that language processing could make ethnic differences disappear. This verifies the importance of language in mathematics, and is consistent with previous findings that language factors, such as well-developed vocabulary knowledge, listening comprehension, and content specific language (i.e., providing direct and explicit instruction with math terminology and concepts), play independent and significant roles in mathematical ability (Rupley and Nichols, 2005).

Although this paper did investigate parental involvement in education or social emotional attitudes, these are variables to be studied in the future. However, the existing literature has shown that the degree of parental education involvement and student social emotional attitudes are related to socioeconomic status (Liu et al., 2015, in Chinese version). As we limited the socioeconomic impact on the results through the balanced selection of participants, the impact of these two variables might also have been controlled for. Nevertheless, for a complete accounting, we should add social factors such as socioeconomic status, educational resources, parental involvement, and social emotion perspective to future research. These four variables have been shown to be related to ethnic differences in academic performance (Marks, 2005; Yan and Lin, 2005; Brinbaum and Cebolla-Boado, 2007; Lee, 2012; Brandmiller et al., 2020; Triventi et al., 2021). The impact of language on mathematic should be re-examined after strictly controlling these variables.

Our results can help improve mathematics education for ethnic minorities. In the future, mathematics education for minority students should focus on the consistency between the language used when teaching mathematics and the official language. Language, especially mathematical language, is a significant predictor of children’s numeracy performance (Purpura and Reid, 2016), and we can consider strengthening Chinese language ability and improving the Chinese language level of minorities, which will help them achieve more academically. Therefore, the next research plan is to carry out an intervention study that employs verbalized mathematics.

Moreover, language intervention might also be useful to improve mathematical ability for majority group students. The difference in language ability can explain not only the ethnic differences in mathematical abilities between minority group students and majority group students, but also the individual differences in mathematical abilities among majority students (e.g., Singer et al., 2019; Peng et al., 2020; Wang et al., 2020; Lin et al., 2021). Previous studies usually paid attention to the effect of mathematical language training on mathematics performances (e.g., Gibson et al., 2020; Purpura et al., 2021), the intervention effect of pure natural language itself should also be into our sight in future.

## Data Availability Statement

The original contributions presented in this study are included in the article/supplementary material, further inquiries can be directed to the corresponding author/s.

## Ethics Statement

The studies involving human participants were reviewed and approved by the Beijing Normal University. Written informed consent to participate in this study was provided by the participants’ legal guardian/next of kin.

## Author Contributions

JC and XZ designed and performed the research. JC, HD, and LL analyzed the data. JC and LL wrote the manuscript. JC, LL, ZC, and XZ revised the manuscript. All authors contributed to the article and approved the submitted version.

## Conflict of Interest

The authors declare that the research was conducted in the absence of any commercial or financial relationships that could be construed as a potential conflict of interest.

## Publisher’s Note

All claims expressed in this article are solely those of the authors and do not necessarily represent those of their affiliated organizations, or those of the publisher, the editors and the reviewers. Any product that may be evaluated in this article, or claim that may be made by its manufacturer, is not guaranteed or endorsed by the publisher.

## Footnotes

1
www.dweipsy.com/lattice
